# Association of the lymphocyte-to–C-reactive protein ratio with long-term mortality in hospitalized older adults with severe dysphagia

**DOI:** 10.3389/fnut.2025.1750904

**Published:** 2026-02-04

**Authors:** Xuejin Zhong, Dongjie Huang, Yuling Chen, Zeru Chen, Xiaomi Chen, Shuyue Zhou, Yutong Lu, Nuoyan Huang, Xiaoling Wu, Haiwei Chen, Mingdi Chen

**Affiliations:** The Second Affiliated Hospital of Guangdong Medical University, Zhanjiang, Guangdong, China

**Keywords:** dysphagia, elderly, immunonutrition, inflammation, lymphocyte-to–C-reactive protein ratio, mortality

## Abstract

**Background:**

Dysphagia is highly prevalent among older adults and is strongly associated with malnutrition, chronic inflammation, and multiple comorbidities, all of which contribute to increased mortality. The lymphocyte-to–C-reactive protein ratio (LCR) has recently gained attention as an immunonutritional biomarker, yet its prognostic relevance in elderly individuals with dysphagia remains unclear. This study examined whether LCR is associated with long-term all-cause mortality in this population.

**Methods:**

This study used a retrospective cohort design involving elderly individuals with dysphagia who underwent either percutaneous endoscopic gastrostomy or total parenteral nutrition from 2014 to 2017. The LCR was converted to its natural logarithmic form and grouped according to its quartile distribution. Survival was analyzed through time-to-event methods, including Kaplan–Meier estimation and multivariable Cox regression. The shape of the association was explored using restricted cubic spline modeling, and several additional analyses were carried out to evaluate the stability of the findings.

**Results:**

The analytic sample comprised 248 older adults, whose mean age was 83.0 years. Survival differed significantly across Ln-LCR quartiles, and higher Ln-LCR values were consistently associated with lower mortality risk. In adjusted Cox models, each one-unit increase in Ln-LCR was associated with a lower hazard of all-cause mortality. Quartile analyses demonstrated a clear graded pattern, and spline models indicated a predominantly linear inverse association, with substantially elevated risk at Ln-LCR values below −1.99. Sensitivity analyses supported the stability of these findings.

**Conclusion:**

Ln-LCR was independently associated with long-term all-cause mortality risk in this cohort and may serve as a convenient prognostic marker for clinical risk stratification. Lower Ln-LCR values may help identify individuals at particularly high risk.

## 1 Introduction

Swallowing is a complex sensorimotor activity that relies on the coordinated function of multiple muscle groups to move ingested material from the oral cavity to the stomach while maintaining airway protection and minimizing pharyngeal residue (1). Disturbances in any part of this mechanism can lead to dysphagia (2). This condition is frequently observed among older adults and often accompanies neurological disorders such as stroke, Parkinson’s disease, and Alzheimer’s disease (3). Dysphagia contributes to serious health consequences, including malnutrition, dehydration, reduced quality of life, and aspiration pneumonia (4), all of which increase the likelihood of mortality. As populations age, the clinical and societal burden associated with dysphagia continues to expand. Despite its impact, dysphagia is commonly under-recognized or insufficiently diagnosed in frail or advanced-age individuals, highlighting the importance of early identification of high-risk patients.

A variety of inflammation- and nutrition-related biomarkers have been examined for their role in evaluating risks associated with dysphagia. Measures such as serum albumin and the C-reactive protein-to-albumin ratio (CAR) have been linked to the presence or severity of dysphagia (5, 6). However, markers capable of reliably predicting overall survival in individuals with dysphagia have not been clearly established. Given that many affected patients face multiple comorbidities, compromised nutritional status, and persistent inflammatory responses, dependable prognostic indicators are essential for improving preventive strategies and informing timely clinical management.

The lymphocyte-to-C-reactive protein ratio (LCR) has gained attention as a composite biomarker that reflects both inflammatory activity and immune function. Its prognostic relevance has been demonstrated across a wide range of conditions, including malignant diseases, chronic liver disorders, and infectious illnesses such as COVID-19 (7–9). C-reactive protein is a major acute-phase reactant that markedly increases during inflammatory activation and serves as an indicator of inflammation severity (10). Lymphocyte counts provide insight into immune competence and often decline under excessive inflammatory stimulation or immune dysregulation (11). By integrating these two parameters, the LCR offers a broader representation of the interaction between inflammation and immune status (12). Despite its expanding clinical applications, research evaluating its association with mortality in older adults with dysphagia is largely lacking.

To bridge this knowledge gap, this study investigated whether LCR is linked to long-term survival among older adults with dysphagia in Japan, independently of other clinical factors. Clarifying its prognostic relevance may facilitate earlier identification of high-risk patients and provide valuable guidance for clinical management in this frail population.

## 2 Materials and methods

### 2.1 Data source and study design

This study employed a longitudinal, retrospective cohort design and received ethical approval from the Miyanomori Memorial Hospital Ethics Committee in Japan (13). The cohort consisted of older adults with clinically confirmed dysphagia who initiated nutritional support through either percutaneous endoscopic gastrostomy (PEG) or total parenteral nutrition (TPN) between January 2014 and January 2017. Patients were not considered eligible if they had terminal-stage malignancies, underwent PEG insertion solely for gastric decompression, or had a PEG placed before 2014 (13).

The dataset used in this analysis was obtained from the DATA-DRYAD repository and originally released by Shigenori Masaki and colleagues (13). All information in the dataset had been fully anonymized and was made publicly accessible under the Creative Commons Attribution–NonCommercial (CC BY-NC 4.0) license, ensuring compliance with relevant ethical and data-use requirements (13). The reliability of this dataset has been supported by several independent validation studies. Because this was a secondary analysis of an existing dataset, no *a priori* sample size calculation was performed.

### 2.2 Calculation of LCR, assessment of dysphagia, and outcome definition

Blood samples for laboratory evaluation were collected by trained phlebotomy personnel. The primary exposure variable was LCR, calculated by dividing the lymphocyte count (cells/μL) by the concentration of C-reactive protein (mg/L) (14–16). Because the distribution of raw LCR values was skewed, natural logarithmic transformation (Ln-LCR) was applied for statistical modeling.

Dysphagia was assessed through a multidisciplinary clinical evaluation by physicians, nursing staff, and speech-language pathologists, and was further evaluated by videofluoroscopy (videofluoroscopic swallowing examination) as part of routine clinical care (13). Based on these assessments, all included participants were identified as having severe impairment in swallowing function and required artificial nutritional support via either PEG or TPN. The principal outcome assessed was death from any cause, calculated from the date nutritional support began until the occurrence of death.

### 2.3 Data collection

A range of variables relevant to the study was extracted from the available database records (17–19). Demographic characteristics included age and sex. Nutritional status was documented using the Clinical Frailty Scale, body mass index, type of nutritional support, and recorded nutritional intake. Comorbidity information encompassed diagnoses of cardiovascular disease, neuromuscular disorders, ischemic heart disease, chronic heart failure, chronic pulmonary disease, chronic liver disease, and chronic kidney disease. In addition, laboratory measurements obtained within 7 days prior to the initiation of nutritional support—such as serum albumin, total lymphocyte count, C-reactive protein, total cholesterol, and hemoglobin—were included for analysis.

### 2.4 Statistical analysis

The cohort was divided into four levels based on the distribution of Ln-LCR values. For descriptive analyses, continuous variables were summarized using either mean-based or median-based metrics depending on their distributional characteristics, while categorical variables were reported as absolute numbers accompanied by proportions. Comparisons across groups employed *χ*^2^ tests for categorical measures. Continuous variables were examined using parametric or non-parametric methods—specifically, the independent-sample *t* test or the Mann–Whitney *U* test—according to data symmetry.

Survival patterns were explored by plotting Kaplan–Meier functions, and differences among Ln-LCR categories were evaluated using the log-rank statistic. Covariates included in multivariable Cox models were prespecified *a priori* based on clinical relevance and prior literature, guided by a conceptual confounding framework (17–19). Only baseline variables measured prior to initiation of nutritional support were considered for adjustment. Model 1 was unadjusted; Model 2 adjusted for age and sex; and Model 3 further adjusted for nutritional status and major comorbidities that could plausibly relate to both Ln-LCR and mortality risk (e.g., body mass index, energy intake, nutrition modality, and comorbid conditions).

The prognostic contribution of Ln-LCR was then assessed by fitting Cox proportional hazards models. Ln-LCR was incorporated both as a continuous measure and as a quartile-based variable. The proportional hazards (PH) assumption for the Cox models was formally assessed using a Schoenfeld residual–based test and visual inspection of the scaled Schoenfeld residual plots. No evidence of PH violation was detected (global test *p* = 0.235), including for Ln-LCR (*p* = 0.807) (Supplementary Table S9); therefore, standard Cox proportional hazards models were considered appropriate. If any PH violation had been observed, we planned to apply an extended Cox approach (e.g., stratified Cox models or time-varying coefficients) as a sensitivity analysis. To evaluate potential dose–response tendencies, the midpoint of each quartile was treated as a continuous term in the model. Restricted cubic spline modeling was applied to visualize possible deviations from linearity, and likelihood ratio tests were used to identify whether the relationship between Ln-LCR and mortality varied across predefined patient subgroups.

A series of complementary analyses was undertaken to determine whether the main results were stable under different analytical conditions. First, incomplete laboratory information was supplemented using a multivariate imputation approach based on chained equations (20), generating five completed datasets. Second, the analyses were repeated after excluding patients who did not live beyond the first 30 days of hospitalization to minimize the influence of early deaths. Third, the Clinical Frailty Scale was incorporated into the fully adjusted Cox model to assess whether frailty altered the association of interest. To further mitigate potential reverse causation (i.e., low LCR reflecting severe acute illness or an imminent terminal condition at baseline), we additionally performed time-lag and restriction sensitivity analyses. Specifically, we conducted landmark (time-lag) analyses using 90-day and 180-day landmarks by restricting the cohort to individuals who survived beyond each landmark time point and re-estimating post-landmark mortality associations using baseline Ln-LCR quartiles. We also repeated analyses after excluding patients with aspiration pneumonia (Asp) and after excluding participants with extreme CR*p* values (defined *a priori*; see Supplementary materials) to reduce the influence of acute/severe inflammatory states.

Furthermore, the prognostic ability of LCR was examined in comparison with several commonly used inflammatory or nutritional markers, including TLC, CRP, and CAR, by evaluating their discriminatory performance through receiver operating characteristic analysis and corresponding AUC estimates. All computations and modeling procedures were performed with R (version 4.2.0), supplemented by analyses conducted in EmpowerStats and the Free Statistics software environment. Statistical significance was determined using two-tailed tests, with values below 0.05 interpreted as indicative of meaningful differences.

## 3 Results

### 3.1 Baseline characteristics on admission of the 248 patients with dysphagia

Of 253 dysphagic inpatients initially identified at Miyaginomori Memorial Hospital, 5 were excluded because lymphocyte data were incomplete, leaving 248 patients for the final analysis. The mean age of these patients was 83.0 ± 9.3 years, and 151 (60.9%) were male. Nutritional support was provided via PEG in 180 patients and via TPN in 68 patients. The clinical and laboratory findings at admission are summarized in Table 1.

**Table 1:** Baseline characteristics of patients with dysphagia.

Variables	Total (*n* = 248)	Q1 (≤ −3.42)	Q2 (−3.42 to −2.00)	Q3 (−2.00 to −0.73)	Q4 (−0.73 to 3.27)	*p*-value
	(*n* = 248)	(*n* = 62)	(*n* = 62)	(*n* = 62)	(*n* = 62)	
Age (years), Mean ± SD	83.0 ± 9.3	85.1 ± 7.0	83.6 ± 8.1	82.3 ± 11.2	81.3 ± 10.1	0.121
Gender, *n* (%)						0.223
Female	97 (39.1)	29 (46.8)	26 (41.9)	24 (38.7)	18 (29)	
Male	151 (60.9)	33 (53.2)	36 (58.1)	38 (61.3)	44 (71)	
TPN, *n* (%)	68 (27.4)	24 (38.7)	21 (33.9)	17 (27.4)	6 (9.7)	**0.002**
PEG, *n* (%)	180 (72.6)	38 (61.3)	41 (66.1)	45 (72.6)	56 (90.3)	**0.002**
CVD, *n* (%)	132 (53.2)	24 (38.7)	33 (53.2)	36 (58.1)	39 (62.9)	**0.043**
Dementia, *n* (%)	100 (40.3)	32 (51.6)	29 (46.8)	22 (35.5)	17 (27.4)	**0.026**
ND, *n* (%)	14 (5.6)	5 (8.1)	1 (1.6)	0 (0)	8 (12.9)	**0.003**
Asp., *n* (%)	93 (37.5)	30 (48.4)	32 (51.6)	18 (29)	13 (21)	**<0.001**
IHD, *n* (%)	44 (17.7)	16 (25.8)	11 (17.7)	10 (16.1)	7 (11.3)	0.200
CHF, *n* (%)	103 (41.5)	37 (59.7)	27 (43.5)	23 (37.1)	16 (25.8)	**0.002**
CLD, *n* (%)	18 (7.3)	6 (9.7)	9 (14.5)	1 (1.6)	2 (3.2)	**0.023**
CKD, *n* (%)	52 (21.0)	16 (25.8)	13 (21)	17 (27.4)	6 (9.7)	0.066
ALB (g/dl), Mean ± SD	3.1 ± 0.6	2.8 ± 0.6	2.9 ± 0.5	3.2 ± 0.5	3.6 ± 0.5	**<0.001**
TLC (ul), Mean ± SD	1,334.3 ± 708.3	868.3 ± 465.8	1,322.5 ± 847.6	1,521.3 ± 641.5	1,625.1 ± 584.8	**<0.001**
TC (mg/dl), Mean ± SD	156.2 ± 40.1	151.1 ± 43.3	149.6 ± 32.3	159.8 ± 45.8	164.4 ± 36.1	0.144
Hemoglobin (g/dl), Mean ± SD	11.0 ± 2.0	10.0 ± 1.9	10.9 ± 1.8	11.0 ± 2.2	12.0 ± 1.7	**<0.001**
CRP (mg/l), Median (IQR)	9.9 (3.0, 32.6)	55.9 (40.8, 73.8)	17.6 (12.6, 25.3)	5.2 (4.0, 8.1)	1.3 (0.6, 2.2)	**<0.001**
BMI, Mean ± SD	19.2 ± 3.3	19.1 ± 3.5	18.5 ± 2.9	20.0 ± 3.9	19.3 ± 2.8	0.075
Kcal/day, Mean ± SD	917.6 ± 187.1	886.4 ± 236.2	895.8 ± 166.1	925.2 ± 174.9	962.9 ± 155.6	0.097
Oral intake recovery, *n* (%)	14 (5.6)	2 (3.2)	2 (3.2)	3 (4.8)	7 (11.3)	0.216
Status, *n* (%)						**<0.001**
Alive	114 (46.0)	17 (27.4)	22 (35.5)	32 (51.6)	43 (69.4)	
Death	134 (54.0)	45 (72.6)	40 (64.5)	30 (48.4)	19 (30.6)	
LCR, Median (IQR)	0.1 (0.0, 0.5)	0.0 (0.0, 0.0)	0.1 (0.0, 0.1)	0.2 (0.2, 0.3)	1.2 (0.7, 2.4)	**<0.001**

CVD, cerebrovascular diseases; ND, neuromuscular diseases; Asp., previous history of aspiration pneumonia; IHD, ischemic heart diseases; CHF, Chronic Heart Failure; CLD, Chronic Lung Diseases; CKD, Chronic Kidney Diseases; ALB, serum albumin; TLC, Total Lymphocyte Count; TC, Total Cholesterol; CRP, C-reactive Protein.

*P*-value less than 0.05 is expressed in bold.

**Figure 1:**
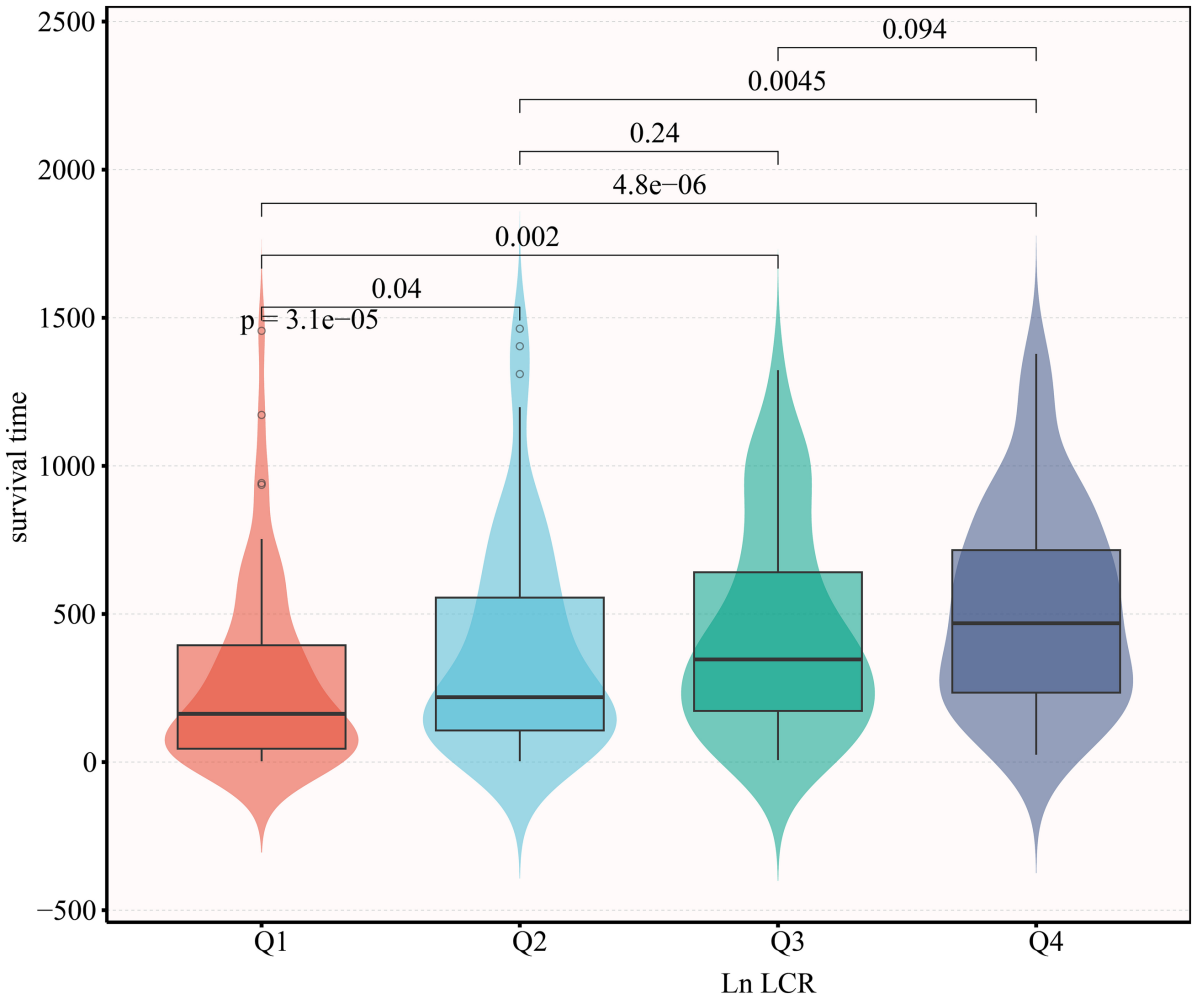
Survival time distribution by ln-transformed LCR index quartiles. Combined boxplot (median, interquartile range, whiskers: min–max excluding outliers) and violin plot (kernel density estimation) demonstrating significant differences in survival time (days) across quartiles of natural log-transformed LCR index. Q1 represents the lowest ln-LCR quartile (red), Q2 (light blue), Q3 (green), and Q4 the highest quartile (dark blue). Survival time progressively increased with ascending LCR quartiles (*p* < 0.001 for trend), with pairwise comparisons showing significantly shorter survival in Q1 versus Q2 (*p* = 3.1 × 10^−5^), Q3 (*p* = 0.002), and Q4 (*p* = 4.8 × 10^−6^). All statistical comparisons performed using Kruskal–Wallis with Dunn’s post-hoc.

On the basis of Ln-LCR quartiles, the cohort was separated into four groups. As illustrated in Figure 1, survival time differed significantly among these four Ln-LCR groups (*p* < 0.05). With increasing Ln-LCR, serum albumin, total lymphocyte count, and hemoglobin levels rose in a graded manner (all *p* < 0.001), whereas CRP levels showed a consistent decline (*p* < 0.001). In addition, the prevalence of comorbid conditions, including CVD, severe dementia, neuromuscular diseases (ND), previous history of aspiration pneumonia (Asp), CHF, and CLD, differed significantly among the Ln-LCR groups (all *p* < 0.05).

### 3.2 Overall mortality across ln-LCR groups

**Figure 2:**
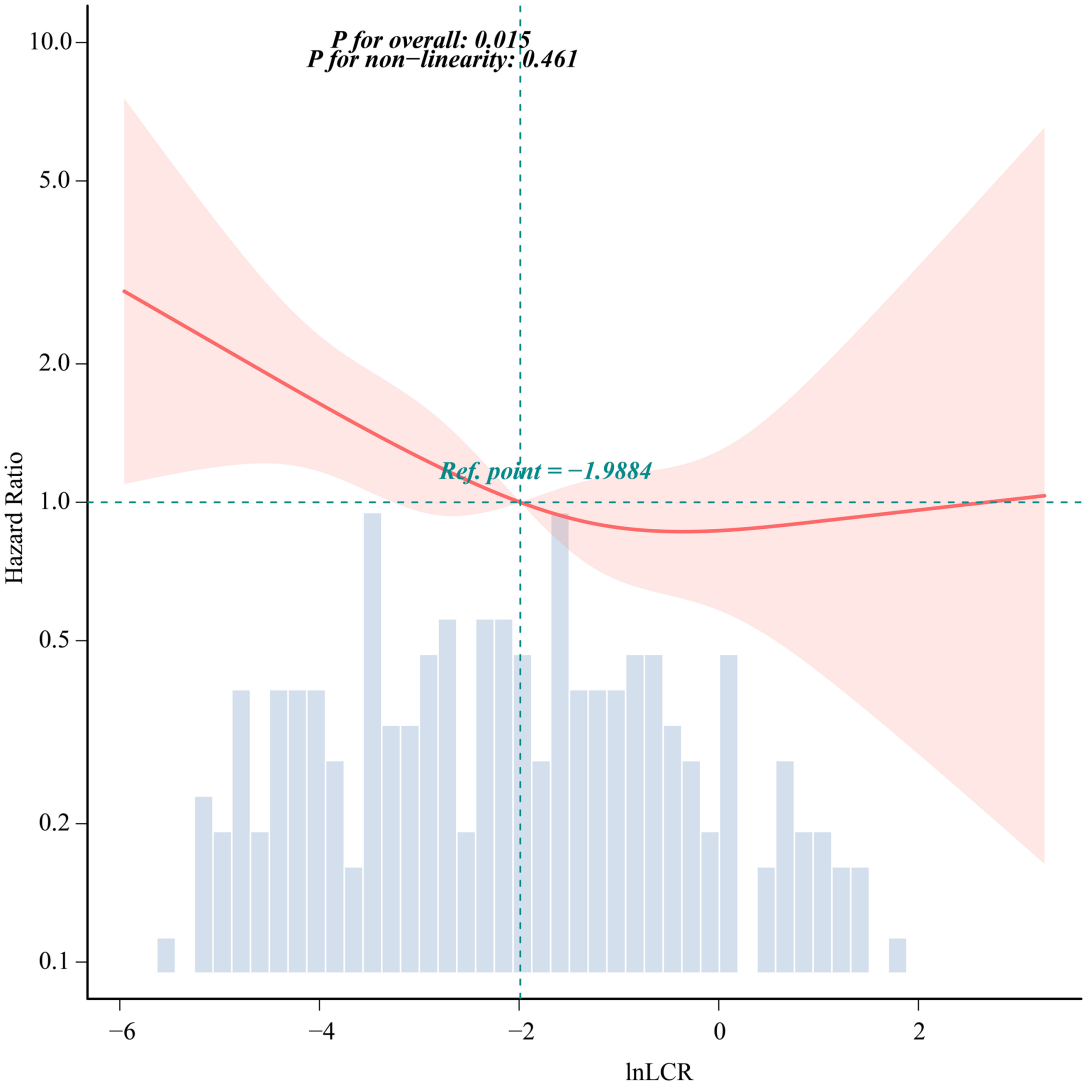
Kaplan–Meier survival analysis stratified by ln-LCR quartiles. Survival probability (%) according to quartiles of natural log-transformed LCR index: Q1 (teal; ≤ −3.42), Q2 (grass green; −3.42 to −2.00), Q3 (dark blue; −2.00 to −0.73), Q4 (pink; −0.73 to 3.27). Log-rank test revealed significantly different survival curves (*p* < 0.0001), with Q1 (lowest ln-LCR) exhibiting the poorest survival. Lower table indicates number at risk at 0, 500, and 1,000-day intervals.

Figure 2 displays the Kaplan–Meier survival estimates by Ln-LCR quartiles. Once the cohort was stratified into four Ln-LCR categories, the corresponding survival curves separated soon after the start of follow-up and remained clearly ordered throughout the observation period, with Q4 showing the most favorable and Q1 the poorest survival profile. When median survival was defined as the time point at which 50% of patients were still alive, the estimated values for Q1, Q2, Q3, and Q4 were approximately 176, 328, 578, and 864 days, respectively. This pattern indicates a progressively longer survival with increasing Ln-LCR quartiles (*P* for trend < 0.0001).

### 3.3 Multivariable cox regression and dose–response relationship between ln-LCR and all-cause mortality

In Table 2, the risk of all-cause death is compared across Ln-LCR quartiles, using the lowest quartile (Q1) as the reference category. Overall, patients in higher Ln-LCR strata tended to have better survival. The hazard ratio for Q2 was slightly below 1 in all three models but did not reach statistical significance. In contrast, Q3 and Q4 showed clearly reduced risks of death, with most hazard ratios falling between 0.45 and 0.52 for Q3 and between 0.24 and 0.52 for Q4. These findings indicate that moderately and markedly elevated Ln-LCR levels are associated with roughly a 50% lower mortality risk compared with Q1. Trend tests across quartiles yielded *p*-values of 0.006 or lower in every model, supporting a graded decline in mortality risk with increasing Ln-LCR.

**Table 2:** Association between natural log-transformed LCR index and survival outcomes across multivariable models.

Variable	Model 1		Model 2		Model 3	
	HR (95% CI)	*P* value	HR (95% CI)	*P* value	HR (95% CI)	*P* value
Ln-LCR continuous	0.75 (0.67–0.82)	<0.001	0.77 (0.7–0.86)	<0.001	0.84 (0.75–0.95)	0.004
Ln-LCR quartiles						
Q1	1.0 [Ref]		1.0 [Ref]		1.0 [Ref]	
Q2	0.68 (0.45–1.05)	0.08	0.77 (0.5–1.18)	0.222	0.68 (0.43–1.06)	0.091
Q3	0.45 (0.28–0.71)	0.001	0.52 (0.33–0.83)	0.006	0.50 (0.30–0.82)	0.007
Q4	0.24 (0.14–0.42)	<0.001	0.30 (0.18–0.52)	<0.001	0.52 (0.29–0.93)	0.028
*P-*trend	0.64 (0.54–0.75)	<0.001	0.68 (0.58–0.8)	<0.001	0.78 (0.65–0.93)	0.006

Model 1: no covariates were adjusted.

Model 2: Gender, age were adjusted.

Model 3: Gender, age, BMI, Kcal/day, CVD, dementia, ND, Asp., IHD, CHF, CLD, CKD, NCVC, PEG, oral intake recovery.

CVD, cerebrovascular diseases; ND, neuromuscular diseases; Asp., previous history of aspiration pneumonia; IHD, ischemic heart diseases; CHF, Chronic Heart Failure; CLD, Chronic Lung Diseases; CKD, Chronic Kidney Diseases; ALB, serum albumin; TLC, Total Lymphocyte Count; TC, Total Cholesterol; CRP, C-reactive Protein; NCVC, Non-tunneled Central Venous Catheters; PEG, Percutaneous Endoscopic Gastrostomy; HR, hazard ratio; CI, confidence interval; Ref reference.

When Ln-LCR was handled as a continuous exposure, a similar inverse pattern was observed. In the crude Cox model (model 1), each 1-unit rise in Ln-LCR corresponded to an approximate 25% reduction in all-cause mortality (HR 0.75, 95% CI 0.67–0.82, *p* < 0.001). Adjustment for sex and age (model 2) resulted in an HR of 0.77 (95% CI 0.70–0.86, *p* < 0.001). After additional control for BMI, energy intake and comorbid conditions (model 3), the association was attenuated but remained statistically significant (HR 0.84, 95% CI 0.75–0.95, *p* = 0.004).

**Figure 3:**
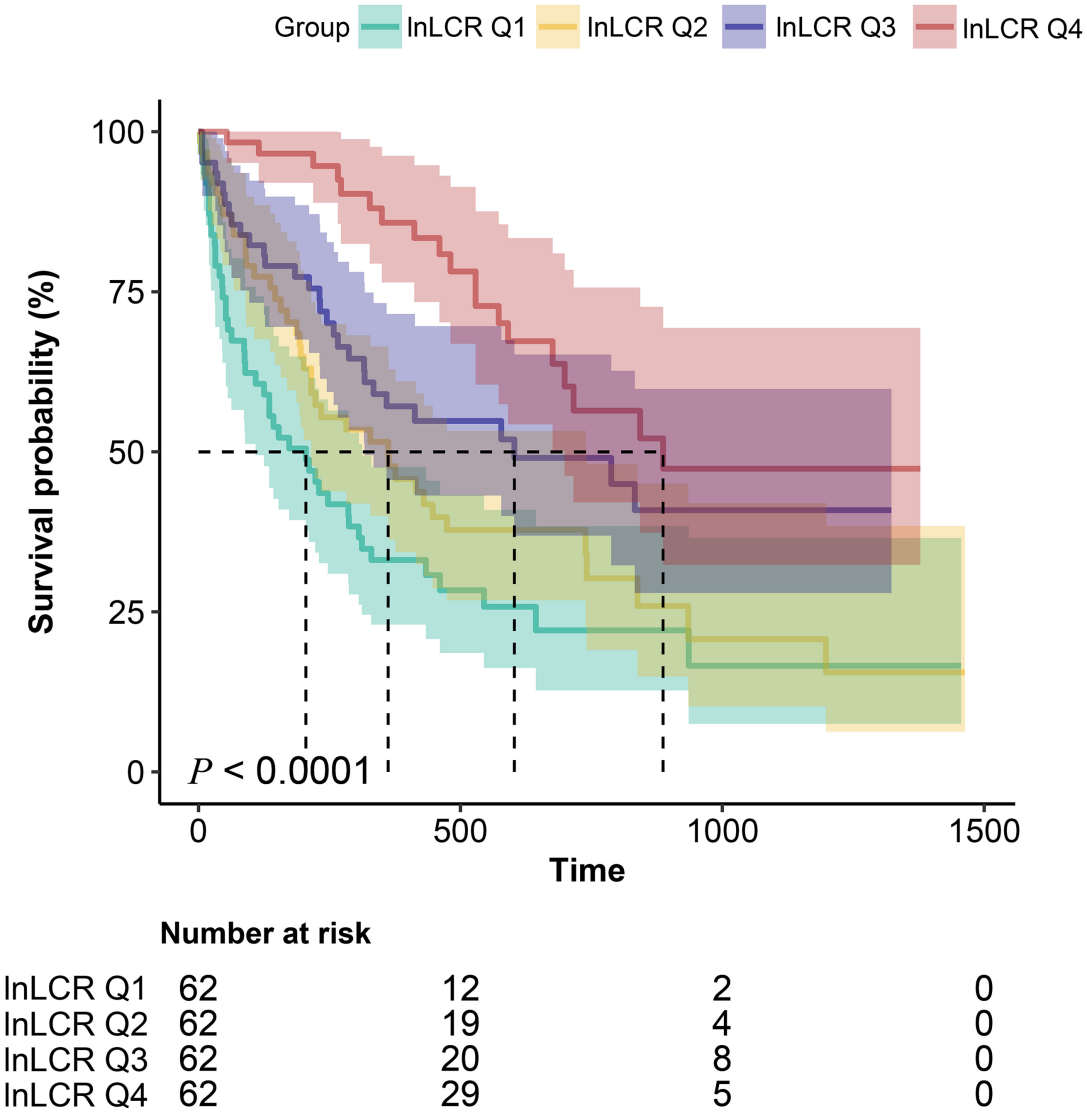
Restricted cubic spline analysis of Ln-LCR index association with overall survival.

To further explore the dose–response pattern, a fully adjusted Cox model with restricted cubic splines was fitted (Figure 3). The global test confirmed a significant overall association between Ln-LCR and all-cause mortality (*P* for overall = 0.015), while no clear deviation from linearity was detected (*P* for non-linearity = 0.461). Taking Ln-LCR = −1.99 as the reference point, values below this threshold were linked to an increased hazard of death, whereas higher Ln-LCR values were associated with hazard ratios hovering around 1.0, with only minor fluctuations.

### 3.4 Subgroup analyses of the association between ln-LCR and all-cause mortality

**Figure 4:**
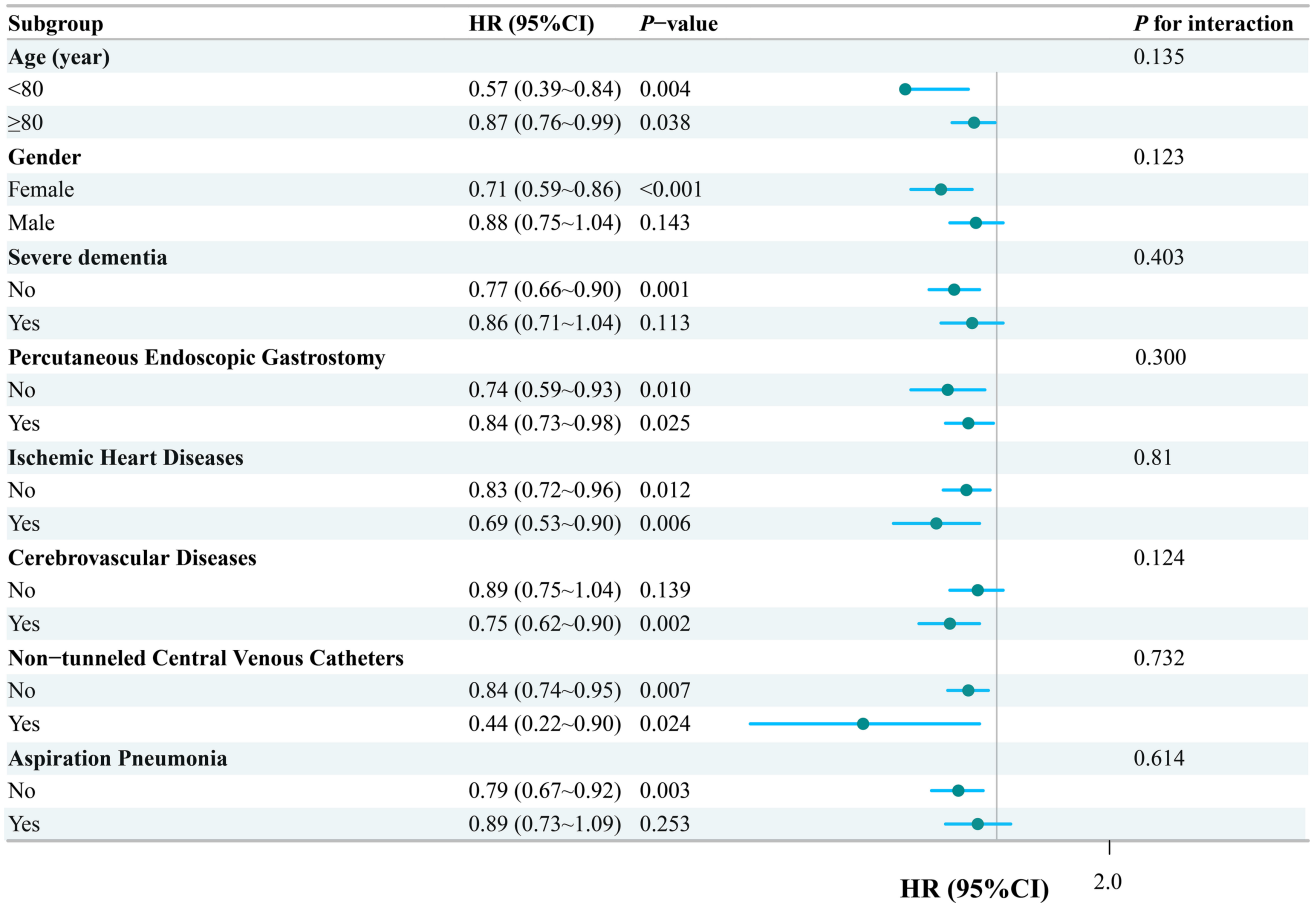
Subgroup analyses of Ln-LCR associated with mortality. Hazard ratios (HRs) were adjusted for age, sex, cerebrovascular diseases, severe dementia, aspiration pneumonia, ischemic heart disease, non-tunneled central venous catheters, percutaneous endoscopic gastrostomy. LCR, lymphocyte-to–C-reactive protein ratio.

In the fully adjusted Cox model, stratified analyses by age, sex, dementia, PEG, IHD, CVD, presence of a non-tunneled central venous catheters (NCVC), and Asp showed that higher Ln-LCR was generally associated with a lower risk of all-cause mortality across subgroups (Figure 4). Although point estimates varied across strata, all *p* values for interaction were > 0.05, providing no evidence that age, sex, or major comorbidities materially modified the association between Ln-LCR and all-cause mortality.

### 3.5 Sensitivity analyses

#### 3.5.1 Multiple imputation of missing lymphocyte data

Missing lymphocyte counts in five patients were handled by MICE approach, yielding five completed datasets whose parameter estimates were pooled according to Rubin’s rules (Supplementary Table S2). In the imputed sample, Ln-LCR entered as a continuous variable in the fully adjusted Cox model was still linked to a lower risk of all-cause mortality (HR 0.83, 95% CI ranging from 0.74 to 0.93), and the quartile analysis continued to show a graded decline in risk from Q1 to Q4 (*P* for trend = 0.002). These findings indicate that the small amount of missing lymphocyte data had only a limited impact on the overall conclusions.

#### 3.5.2 Exclusion of early in-hospital deaths

To reduce potential distortion by patients in a terminal phase at admission, the analysis was repeated after restricting the cohort to individuals who survived more than 30 days after hospitalization and refitting the multivariable Cox model (Supplementary Table S3). In this restricted population, the hazard estimate for Ln-LCR as a continuous variable was approximately 0.85, and the quartile models again showed progressively lower mortality with increasing Ln-LCR, closely mirroring the primary results. These observations suggest that early in-hospital deaths does not materially alter the inverse relationship between Ln-LCR and mortality.

#### 3.5.3 Landmark (time-lag) analyses to mitigate reverse causation

To further reduce potential reverse causation (i.e., low LCR reflecting imminent death or a terminal condition at baseline), we conducted landmark (time-lag) analyses using 90-day and 180-day landmarks (Supplementary Tables S7–S8). In the 90-day landmark analysis, the inverse association between baseline Ln-LCR quartiles and subsequent mortality remained evident among patients who survived beyond 90 days: compared with Q1, Q3 and Q4 were associated with lower post-landmark mortality (Q3 vs. Q1: HR 0.538, 95% CI 0.293–0.986, *p* = 0.0449; Q4 vs. Q1: HR 0.401, 95% CI 0.215–0.749, *p* = 0.0042), with a significant dose–response trend (*P* for trend < 0.001). In the 180-day landmark analysis, the overall trend across quartiles remained statistically significant after 180 days (*P* for trend = 0.0217), while the point estimate for Q4 vs. Q1 remained protective but with wider confidence intervals (HR 0.523, 95% CI 0.261–1.048, *p* = 0.0677), likely due to fewer events in longer-term survivors.

#### 3.5.4 Exclusion of aspiration pneumonia (asp)

To reduce the influence of acute infection/inflammation-related severity, we repeated the analyses after excluding patients with aspiration pneumonia (Asp) (Supplementary Table S10). In the fully adjusted model, Ln-LCR remained inversely associated with all-cause mortality (HR per 1-unit increase in Ln-LCR = 0.81, 95% CI 0.69–0.96; *p* = 0.012). Quartile analyses showed a similar graded pattern, with Q4 having a significantly lower mortality risk than Q1 (HR 0.34, 95% CI 0.14–0.83; *p* = 0.017; *P* for trend = 0.013).

#### 3.5.5 Exclusion of extreme CRP values

We further repeated the analyses after excluding participants with extreme CRP values (definition provided in Supplementary Table S11) to reduce the potential impact of acute severe inflammation. In the fully adjusted model, the association remained directionally consistent but was attenuated (HR per 1-unit increase in Ln-LCR = 0.82, 95% CI 0.66–1.03; *p* = 0.088). The overall trend across quartiles remained statistically significant (*P* for trend = 0.03), although individual quartile comparisons were less precise.

#### 3.5.6 Inclusion of CFS in the multivariable cox model

Because high levels of frailty and systemic inflammation often coexist in older inpatients with dysphagia, potential collinearity involving the Clinical Frailty Scale (CFS) was examined using generalized variance inflation factors and condition indices (Supplementary Table S1). The GVIF^(1/(2 × Df)) for CFS was close to 1, indicating no relevant multicollinearity.

When CFS was then added to the fully adjusted Cox model (Supplementary Table S4), the hazard ratio for Ln-LCR remained 0.84, with an absolute change of less than 0.01 compared with the main model, and the direction and statistical significance of the association were unchanged. Thus, the influence of the frailty score on the estimated association between Ln-LCR and all-cause mortality can be regarded as negligible.

#### 3.5.7 Comparison of the predictive performance of ln-LCR with other inflammatory/nutritional indices

The prognostic performance of Ln-LCR was further compared with that of TLC, CRP and lnACR (Supplementary Table S5). In multivariable model 3, Ln-LCR and TLC, when treated as continuous variables, were both significantly related to all-cause mortality, whereas the continuous model for CRP yielded a *p* value of 0.071 and did not reach statistical significance. Ln-ACR was associated with mortality when modelled continuously (HR 1.14, 95% CI ranging from 1.01 to 1.29, *p* = 0.040), but the association weakened when Ln-ACR was analyzed in quartiles (*P* for trend = 0.084). Compared with TLC, a one-unit increase in Ln-LCR corresponded to a larger deviation of the hazard ratio from 1.0, indicating a steeper mortality gradient across Ln-LCR values.

ROC analyses provided additional support for this pattern: the area under the curve for Ln-LCR was 0.654 (95% CI ranging from 0.584 to 0.723), which was higher than that for Ln-ACR (0.642), CRP (0.636) and TLC (0.630) (Supplementary Table S6 and Figure S1). Among the four markers, Ln-LCR therefore showed the best overall discriminative ability for predicting all-cause mortality.

## 4 Discussion

This study is the first to systematically examine the prognostic association between Ln-LCR and long-term all-cause mortality in hospitalized older adults with severe dysphagia requiring PEG or TPN. In this cohort, higher Ln-LCR was independently associated with a lower risk of all-cause mortality. Given the retrospective observational design, these findings should be interpreted as prognostic associations rather than evidence of causality or treatment benefit. In fully adjusted models, each one-unit increase in Ln-LCR was associated with a 16% lower hazard of death, and the overall inverse trend across Ln-LCR quartiles was consistent. Although the Q2–Q1 comparison did not reach statistical significance in several models (with confidence intervals crossing 1.0), this likely reflects modest differences between adjacent categories and the reduced efficiency of categorizing a continuous exposure; therefore, interpretation was based primarily on the overall dose–response pattern and the continuous analyses. Ln-LCR also showed comparatively better discriminative performance than several commonly used inflammatory/nutritional indices.

In recent years, composite indicators derived from inflammation- and nutrition-related blood biomarkers—such as the granulocyte-to-lymphocyte ratio, CAR, and prognostic nutritional index—have garnered significant attention for their predictive value in diseases including liver cancer, colorectal cancer, lung cancer, and myocardial infarction (21–25). Among these, CAR is regarded as an effective indicator reflecting the balance between nutrition and inflammation (26). Previous studies have also shown a positive correlation between CAR and mortality in elderly Japanese patients with dysphagia (6), which is consistent with our findings. Meanwhile, in this study, ROC curve analysis revealed that LCR had superior predictive performance for elderly patients with dysphagia compared to CAR. This may be because, in extremely frail elderly populations, lymphocyte count is more sensitive than albumin in reflecting short-term nutritional depletion and immune dysfunction (27, 28). Albumin has a relatively long half-life (approximately 20 days) and is significantly influenced by liver function and fluid status (29, 30), whereas lymphocyte counts change more rapidly, providing a more timely reflection of the body’s stress and exhaustion state (31). Therefore, LCR, which is centered on lymphocytes, may capture the immediate risk in this specific population more effectively than CAR, which is centered on albumin.

The inverse relationship between LCR and mortality risk in elderly dysphagia patients may be mechanistically rooted in the vicious cycle of “inflammation–malnutrition–immunosuppression.” The elevated mortality in elderly patients with dysphagia is closely related to malnutrition (2, 32, 33), and lymphocytopenia is a key manifestation of this pathological process. A decrease in lymphocyte count not only reflects immunosenescence and thymic involution but also directly indicates immunodeficiency resulting from malnutrition (34, 35). Research has shown that short-term caloric restriction can significantly reduce lymphocyte numbers in mammalian lymphoid tissues (36), suggesting that lymphocytopenia may serve as a sensitive marker of inadequate energy and nutrient intake. Concurrently, elevated C-reactive protein signifies a persistent systemic inflammatory state (37). By promoting the release of pro-inflammatory cytokines such as IL-1, IL-6, IL-18, and tumor necrosis factor-alpha, it acts on the central nervous system to induce anorexia, exacerbating insufficient energy and protein intake (38), while also directly activating catabolic pathways such as the ubiquitin–proteasome system, leading to skeletal muscle protein breakdown and the development of sarcopenia (32, 39). In dysphagia patients, pre-existing intake difficulties and inflammation-driven metabolic disturbances interact, forming a self-reinforcing vicious cycle: the inflammatory state (elevated CRP) exacerbates malnutrition and immunosuppression, while immune dysfunction (lymphocytopenia) increases infection risk, triggering more intense inflammatory responses. The persistence of this cycle continuously depletes the patient’s physiological reserves, ultimately leading to multi-organ functional decline and frailty, significantly reducing tolerance to various stressors and markedly increasing mortality risk (40).

Notably, in the analysis of the association between Ln-LCR and mortality risk, although the trend test supported a linear relationship, the RCS curve indicated a change point near Ln-LCR = −1.99. When lnLCR was below this value, mortality risk increased sharply with decreasing Ln-LCR, reflecting a severe decompensated state where even minor additional stressors could significantly affect prognosis. When Ln-LCR was above this level, the risk curve flattened, with the HR approaching 1.0 and exhibiting less fluctuation, suggesting that the immune–inflammatory system may have entered a relatively stable compensatory phase, where further improvements in the indicator provided limited marginal protective effects (41). Although this finding does not negate the overall linear trend, it indicates that the risk gradient of Ln-LCR differs across its range, offering a more nuanced reference for its clinical interpretation.

Our study has clear potential for clinical translation. First, as a derived indicator based on routine blood tests, LCR offers the prominent advantages of being economical, convenient, and reproducible, making it easy to promote and use across healthcare institutions at all levels. For hospitalized patients with dysphagia, calculating their LCR can help quickly identify high-risk individuals in a state of “high inflammation–low immune nutrition,” thereby alerting clinicians to provide more proactive attention and intervention. Second, although it remains unclear whether actively increasing LCR through interventions (such as nutritional support or anti-inflammatory therapy) can directly improve prognosis, using it as a dynamic monitoring indicator to assess the effectiveness of interventions and the progression of the patient’s condition is undoubtedly a promising research direction.

Strengths of this study include a long follow-up period, comprehensive covariate adjustment, and multiple sensitivity analyses (e.g., multiple imputation and exclusion of early deaths) that supported the robustness of the findings. However, several limitations should be noted. First, this study was a secondary analysis of a retrospective dataset from a single center with a modest sample size (*n* = 248), and no *a priori* sample-size estimation was performed. Second, the cohort was restricted to hospitalized older adults with severe dysphagia requiring PEG or TPN, which may limit generalizability to mild-to-moderate dysphagia or community-dwelling populations. Third, because the original dataset did not capture some clinically important information (e.g., detailed acute illness severity, infection burden, and medication use such as antibiotics or corticosteroids), residual confounding and reverse causation cannot be fully excluded; therefore, the magnitude of the observed association should be interpreted cautiously. To address this concern, we performed additional time-lag and restriction sensitivity analyses, including 90-day and 180-day landmark analyses, exclusion of patients with aspiration pneumonia, and exclusion of extreme CRP values. The inverse association between baseline Ln-LCR and subsequent mortality remained directionally consistent across these analyses, suggesting that the findings are unlikely to be driven solely by very early terminal events or acute severe inflammatory states at baseline. Nevertheless, as an observational study with a single baseline measurement, residual reverse causation cannot be completely ruled out. Fourth, Ln-LCR was assessed only once at baseline prior to initiation of nutritional support, and longitudinal measurements were unavailable, which may introduce exposure misclassification (regression dilution) and attenuate the observed association toward the null. Finally, subgroup analyses should be considered exploratory given the modest sample size and may be underpowered to detect effect modification; therefore, subgroup-specific patterns should be interpreted cautiously to avoid overinterpretation.

Future research directions should include: conducting multicenter, large-scale prospective cohort studies to validate the generalizability of our conclusions; delving deeper into the trajectory of LCR’s dynamic changes over time and its prognostic value; and, based on this, exploring comprehensive treatment strategies aimed at elevating LCR through nutritional and anti-inflammatory interventions, and assessing their actual efficacy in improving clinical endpoints.

## 5 Conclusion

In hospitalized older adults with severe dysphagia, admission Ln-LCR was independently associated with long-term all-cause mortality risk and may be useful as a prognostic marker for early risk stratification. This indicator comprehensively reflects the body’s immunonutritional status and inflammatory load, and its predictive performance surpasses that of previous indicators such as CAR. Furthermore, when Ln-LCR falls within the lower range below −1.99, it may help identify a patient subgroup with significantly elevated mortality risk, warranting greater attention in the clinical management of this population. Future studies should further explore the clinical applicability of this reference range and investigate the feasibility and effectiveness of interventions, such as targeted nutritional support, to increase Ln-LCR levels and thereby improve long-term patient outcomes.

## Data Availability

The original contributions presented in the study are included in the article/Supplementary material, further inquiries can be directed to the corresponding author.
