# Supplementary material for: Association of the lymphocyte-to–C-reactive protein ratio with long-term mortality in hospitalized older adults with severe dysphagia
Source: Front Nutr. 2026 Feb 4;12:1750904. doi: 10.3389/fnut.2025.1750904 (PMC12913077; doi:10.3389/fnut.2025.1750904)
Supplement: Supplementary file 1 [file Table_1.DOCX]

**Supplementary Table S1. Assessment of multicollinearity across covariates.**

| Term1 | GVIF | DF | GVIF^(1/(2*Df)) | Colinearity (0=No, 1=Yes) |
| --- | --- | --- | --- | --- |
| Crude | 1.112 | 1 | 1.054 | 0 |
| Age | 1.322 | 1 | 1.150 | 0 |
| Sex | 1.579 | 1 | 1.257 | 0 |
| CVD | 1.956 | 1 | 1.399 | 0 |
| Dementia | 2.029 | 1 | 1.424 | 0 |
| Asp | 1.617 | 1 | 1.271 | 0 |
| IHD | 1.566 | 1 | 1.251 | 0 |
| ND | 1.374 | 1 | 1.172 | 0 |
| CHF | 1.532 | 1 | 1.238 | 0 |
| CLD | 1.117 | 1 | 1.057 | 0 |
| CKD | 1.548 | 1 | 1.244 | 0 |
| BMI | 1.363 | 1 | 1.168 | 0 |
| Oral | 1.027 | 1 | 1.013 | 0 |
| PEG | 1.895 | 1 | 1.377 | 0 |
| NCVC | 1.736 | 1 | 1.318 | 0 |
| Daily calorie intake | 1.498 | 1 | 1.224 | 0 |
| CFS | 1.129 | 1 | 1.020 | 0 |

Abbreviations: PEG percutaneous endoscopic gastrostomy；TPN total parenteral nutrition；CVD cerebrovascular diseases；Dementia severe dementia；Asp aspiration pneumonia；IHD ischemic heart diseases; Oral oral intake recovery.

**Supplementary Table S2. Association Between LCR Index and Survival Outcomes Across Multivariable Models after MICE.**

| **Variable** | **Total** | **Event(%)** | **Crude Model** | | **Model 3** | |
| --- | --- | --- | --- | --- | --- | --- |
|  |  |  | **HR(95%CI)** | ***P*** | **HR(95%CI)** | ***P*** |
| lnLCR | 253 | 138 (54.5) | 0.75 (0.68~0.82) | <0.001 | 0.83 (0.74~0.93) | 0.002 |
| **Quartiles** |  |  |  |  |  |  |
| Q1 | 63 | 47 (74.6) | 1(Ref) |  | 1(Ref) |  |
| Q2 | 63 | 40 (63.5) | 0.65 (0.42~0.99) | 0.043 | 0.69 (0.44~1.08) | 0.104 |
| Q3 | 63 | 31 (49.2) | 0.42 (0.27~0.67) | <0.001 | 0.46 (0.28~0.76) | 0.002 |
| Q4 | 64 | 20 (64.0) | 0.23 (0.14~0.40) | <0.001 | 0.49 (0.28~0.87) | 0.014 |
| P for trend |  |  |  | <0.001 |  | 0.002 |

Notes: Crude: Unadjusted;

Model 3: Gender, age, BMI, Kcal/day, CVD, dementia, ND, Asp, IHD, CHF, CLD, CKD, NCVC, PEG, oral intake recovery.

Abbreviation: CVD, cerebrovascular diseases; ND, neuromuscular diseases; Asp, previous history of aspiration pneumonia; IHD, ischemic heart diseases; CHF, Chronic Heart Failure; CLD, Chronic Lung Diseases; CKD, Chronic Kidney Diseases; ALB, serum albumin; TLC, Total Lymphocyte Count; TC, Total Cholesterol; CRP, C-reactive Protein; NCVC, Non-tunneled Central Venous Catheters; PRG, Percutaneous Endoscopic Gastrostomy; HR hazard ratio; CI confidence interval; Ref reference.

| **Variable** | **Crude Model** | | **Model 1** | | **Model 2** | | **Model 3** | |
| --- | --- | --- | --- | --- | --- | --- | --- | --- |
|  | **HR(95%CI)** | ***P*** | **HR(95%CI)** | ***P*** | **HR(95%CI)** | ***P*** | **HR(95%CI)** | ***P*** |
| lnLCR | 0.78(0.70~0.86) | <0.001 | 0.80(0.72~0.89) | <0.001 | 0.86(0.77~0.95) | 0.005 | 0.85(0.76~0.95) | 0.005 |
| **Quartiles** |  |  |  |  |  |  |  |  |
| Q1 | 1(Ref) |  | 1(Ref) |  | 1(Ref) |  | 1(Ref) |  |
| Q2 | 0.77(0.49~1.22) | 0.268 | 0.85(0.54~1.33) | 0.470 | 0.76(0.45~1.28) | 0.297 | 0.76(0.47~1.24) | 0.272 |
| Q3 | 0.49(0.29~0.80) | 0.005 | 0.54(0.33~0.90) | 0.017 | 0.63(0.38~1.04) | 0.069 | 0.53(0.31~0.91) | 0.023 |
| Q4 | 0.28(0.16~0.49) | <0.001 | 0.34(0.59~0.83) | <0.001 | 0.41 (0.22~0.74) | 0.003 | 0.62(0.31~0.95) | 0.049 |
| P for trend |  | <0.001 |  | <0.001 |  | 0.002 |  | 0.020 |

**Supplementary Table S3. Associations between the log-transformed LCR index and overall survival in patients with survival of more than 30 days.**

Notes: Crude:

Model 1: no covariates were adjusted

Model 2: Gender, age were adjusted

Model 3: Gender, age, BMI, Kcal/day, CVD, dementia, ND, Asp, IHD, CHF, CLD, CKD, NCVC, PEG, oral intake recovery.

Abbreviation: CVD, cerebrovascular diseases; ND, neuromuscular diseases; Asp, previous history of aspiration pneumonia; IHD, ischemic heart diseases; CHF, Chronic Heart Failure; CLD, Chronic Lung Diseases; CKD, Chronic Kidney Diseases; ALB, serum albumin; TLC, Total Lymphocyte Count; TC, Total Cholesterol; CRP, C-reactive Protein; NCVC, Non-tunneled Central Venous Catheters; PRG, Percutaneous Endoscopic Gastrostomy; HR hazard ratio; CI confidence interval; Ref reference.

**Supplementary Table S4. Additional adjusted for CFS in Multivariable Models.**

| **Variable** | **Total** | **Event(%)** | **Crude Model** | | **Model 3** | |
| --- | --- | --- | --- | --- | --- | --- |
|  |  |  | **HR(95%CI)** | ***P*** | **HR(95%CI)** | ***P*** |
| lnLCR | 248 | 134 (54) | 0.75 (0.67~0.82) | <0.001 | 0.84 (0.75~0.94) | 0.003 |
| **Quartiles** |  |  |  |  |  |  |
| Q1 | 62 | 485(72.6) | 1(Ref) |  | 1(Ref) |  |
| Q2 | 62 | 40 (64.5) | 0.68 (0.45~1.05) | 0.080 | 0.86 (0.54~1.37) | 0.533 |
| Q3 | 62 | 30 (48.4) | 0.45 (0.28~0.71) | 0.001 | 0.53 (0.32~0.88) | 0.014 |
| Q4 | 62 | 19 (30.6) | 0.24 (0.14~0.42) | <0.001 | 0.52 (0.29~0.93) | 0.027 |
| P for trend |  |  |  | <0.001 |  | 0.004 |

Notes: Crude:

Model 1: no covariates were adjusted

Model 2: Gender, age were adjusted

Model 3: Gender, age, BMI, Kcal/day, CVD, dementia, ND, Asp, IHD, CHF, CLD, CKD, NCVC, PEG, CFS, oral intake recovery.

Abbreviation: CVD, cerebrovascular diseases; ND, neuromuscular diseases; Asp, previous history of aspiration pneumonia; IHD, ischemic heart diseases; CHF, Chronic Heart Failure; CLD, Chronic Lung Diseases; CKD, Chronic Kidney Diseases; ALB, serum albumin; TLC, Total Lymphocyte Count; TC, Total Cholesterol; CRP, C-reactive Protein; NCVC, Non-tunneled Central Venous Catheters; PRG, Percutaneous Endoscopic Gastrostomy; HR hazard ratio; CI confidence interval; Ref reference.

**Supplementary Table S5. Associations between four nutrition index and overall survival in patients.**

| **Variable** | **Total** | **Event_%** | **Crude Model** | | **Model 3** | |
| --- | --- | --- | --- | --- | --- | --- |
|  |  |  | **HR (95%CI)** | ***P*** | **HR (95%CI)** | ***P*** |
| **lnLCR** | 253 | 138 (54.5) | 0.75 (0.68~0.82) | <0.001 | 0.83 (0.74~0.93) | 0.002 |
| Q1 | 63 | 47 (74.6) | 1(Ref) |  | 1(Ref) |  |
| Q2 | 63 | 38 (63.5) | 0.65 (0.42~0.99) | 0.043 | 0.69 (0.44~1.08) | 0.104 |
| Q3 | 63 | 32 (49.2) | 0.42 (0.27~0.67) | <0.001 | 0.46 (0.28~0.76) | 0.002 |
| Q4 | 64 | 19 (31.2) | 0.23 (0.14~0.40) | <0.001 | 0.49 (0.28~0.87) | 0.014 |
| P for trend |  |  |  | <0.001 |  | 0.002 |
| **TLC** | 253 | 138 (54.5) | 1.00 (0.99~1.00) | <0.001 | 1.00 (0.99~1.00) | 0.019 |
| Q1 | 63 | 50 (79.4) | 1(Ref) |  | 1(Ref) |  |
| Q2 | 63 | 34 (54.0) | 0.36 (0.23~0.57) | <0.001 | 0.56 (0.35~0.91) | 0019 |
| Q3 | 63 | 28 (44.4) | 0.35 (0.22~0.55) | <0.001 | 0.46 (0.28~0.75) | 0.002 |
| Q4 | 64 | 26 (40.6) | 0.26 (0.16~0.42) | <0.001 | 0.35 (0.21~0.60) | <0.001 |
| P for trend |  |  |  | <0.001 |  | <0.001 |
| **CRP** | 253 | 138 (54.5) | 1.10 (1.05~1.15) | <0.001 | 1.06 (1.00~1.12) | 0.071 |
| Q1 | 63 | 22 (34.9) | 1(Ref) |  | 1(Ref) |  |
| Q2 | 63 | 29 (46.0) | 1.47 (0.85~2.57) | 0.171 | 1.28 (0.71~2.30) | 0.405 |
| Q3 | 63 | 41 (65.1) | 2.56 (1.52~4.30) | <0.001 | 1.20 (0.67~2.16) | 0.534 |
| Q4 | 64 | 46 (71.9) | 3.40 (2.04~5.66) | <0.001 | 1.88 (1.08~3.28) | 0.261 |
| P for trend |  |  |  | <0.001 |  | 0.030 |
| **lnACR** | 253 | 138 (54.5) | 1.33 (1.20~1.48) | <0.001 | 1.14 (1.01~1.29) | 0.040 |
| Q1 | 63 | 20 (31.7) | 1(Ref) |  | 1(Ref) |  |
| Q2 | 63 | 31 (49.2) | 1.89 (1.08~3.32) | 0.027 | 1.19 (0.66~2.15) | 0.563 |
| Q3 | 63 | 42 (66.7) | 3.04 (1.78~5.18) | <0.001 | 1.27 (0.70~2.30) | 0.433 |
| Q4 | 64 | 45 (70.3) | 3.63 (2.14~6.16) | <0.001 | 1.62 (0.98~2.88) | 0.099 |
| P for trend |  |  |  | <0.001 |  | 0.084 |

Notes: Crude:

Model 1: no covariates were adjusted

Model 2: Gender, age were adjusted

Model 3: Gender, age, BMI, Kcal/day, CVD, dementia, ND, Asp, IHD, CHF, CLD, CKD, NCVC, PEG, oral intake recovery.

Abbreviation: CVD, cerebrovascular diseases; ND, neuromuscular diseases; Asp, previous history of aspiration pneumonia; IHD, ischemic heart diseases; CHF, Chronic Heart Failure; CLD, Chronic Lung Diseases; CKD, Chronic Kidney Diseases; ALB, serum albumin; TLC, Total Lymphocyte Count; TC, Total Cholesterol; CRP, C-reactive Protein; NCVC, Non-tunneled Central Venous Catheters; PRG, Percutaneous Endoscopic Gastrostomy; HR hazard ratio; CI confidence interval; Ref reference.

**Supplementary Table S6. Comparison of the predictive ability of four nutrition index for patients.**

| **Test** | **AUC** | **95%CI lower** | **95%CI Upper** | **Specificity** | **Sensitivity** | **Accuracy** | **NPV** | **PPV** |
| --- | --- | --- | --- | --- | --- | --- | --- | --- |
| lnLCR | 0.654 | 0.584 | 0.723 | 0.516 | 0.707 | 0.614 | 0.625 | 0.606 |
| lnCAR | 0.642 | 0.579 | 0.711 | 0.508 | 0.699 | 0.606 | 0.616 | 0.599 |
| CRP | 0.636 | 0.566 | 0.704 | 0.482 | 0.722 | 0.605 | 0.622 | 0.595 |
| TLC | 0.63 | 0.556 | 0.698 | 0.711 | 0.479 | 0.593 | 0.565 | 0.636 |

Abbreviations: CRP, C-reactive Protein; TLC, Total Lymphocyte Count.

**Supplementary Table S7. 90-day landmark (time-lag) analysis of baseline Ln-LCR quartiles and all-cause mortality in elderly patients with dysphagia.**

| **Variable** | **n.total** | **n.event_%** | **Followup.Time** | **crude.OR_95CI** | **crude.P_value** |
| --- | --- | --- | --- | --- | --- |
| Time below the landmark |  |  |  |  |  |
| lnlcrQ41 | 62 | 23 (37.1) | 4275 | 1(Ref) |  |
| lnlcrQ42 | 62 | 10 (16.1) | 5000 | 0.380 (0.181~0.799) | 0.0107 |
| lnlcrQ43 | 62 | 10 (16.1) | 5067 | 0.374 (0.178~0.787) | 0.0095 |
| lnlcrQ44 | 62 | 1 (1.6) | 5366 | 0.036 (0.005~0.263) | 0.0011 |
| Trend.test | 248 | 44 (17.7) | 19708 | 0.473 (0.343~0.650) | <0.001 |
| Time over the landmark |  |  |  |  |  |
| lnlcrQ41 | 37 | 22 (59.5) | 16083 | 1(Ref) |  |
| lnlcrQ42 | 52 | 30 (57.7) | 23243 | 0.978 (0.563~1.697) | 0.9364 |
| lnlcrQ43 | 52 | 20 (38.5) | 26442 | 0.538 (0.293~0.986) | 0.0449 |
| lnlcrQ44 | 56 | 18 (32.1) | 31997 | 0.401 (0.215~0.749) | 0.0042 |
| Trend.test | 197 | 90 (45.7) | 97765 | 0.716 (0.593~0.865) | <0.001 |

**Supplementary Table S8. 180-day landmark (time-lag) analysis of baseline Ln-LCR quartiles and all-cause mortality in elderly patients with dysphagia.**

| **Variable** | **n.total** | **n.event_%** | **Followup.Time** | **crude.OR_95CI** | **crude.P_value** |
| --- | --- | --- | --- | --- | --- |
| **Time below the landmark** |  |  |  |  |  |
| lnlcrQ41 | 62 | 30 (48.4) | 7314 | 1(Ref) |  |
| lnlcrQ42 | 62 | 18 (29) | 8905 | 0.505 (0.281~0.907) | 0.0222 |
| lnlcrQ43 | 62 | 13 (21) | 9475 | 0.349 (0.182~0.670) | 0.0016 |
| lnlcrQ44 | 62 | 2 (3.2) | 10336 | 0.050 (0.012~0.210) | <0.001 |
| Trend.test | 248 | 63 (25.4) | 36030 | 0.482 (0.371~0.626) | <0.001 |
| **Time over the landmark** |  |  |  |  |  |
| lnlcrQ41 | 29 | 15 (51.7) | 14934 | 1(Ref) |  |
| lnlcrQ42 | 39 | 22 (56.4) | 21678 | 1.030 (0.533~1.990) | 0.9293 |
| lnlcrQ43 | 46 | 17 (37) | 25634 | 0.641 (0.320~1.286) | 0.2108 |
| lnlcrQ44 | 54 | 17 (31.5) | 31707 | 0.523 (0.261~1.048) | 0.0677 |
| Trend.test | 168 | 71 (42.3) | 93953 | 0.780 (0.632~0.964) | 0.0217 |

**Supplementary Table S9. Schoenfeld residual–based tests of the proportional hazards assumption for the fully adjusted Cox model (overall follow-up).**

| **Variable** | **chisq** | **df** | **p.value** |
| --- | --- | --- | --- |
| Ln-LCR | 0.059 | 1 | 0.807 |
| PEG | 0.539 | 1 | 0.463 |
| NCVC | 0.904 | 1 | 0.342 |
| age | 0.026 | 1 | 0.872 |
| gender | 0.124 | 1 | 0.724 |
| cerebrovascular_diseases | 0.183 | 1 | 0.669 |
| severe_dementia | 1.645 | 1 | 0.2 |
| neuromuscular_diseases | 0.447 | 1 | 0.504 |
| previous_history_of_aspiration_pneumonia | 1.078 | 1 | 0.299 |
| ischemic_heart_diseases | 1.49 | 1 | 0.222 |
| chronic_heart_failure | 1.503 | 1 | 0.22 |
| chronic_lung_diseases | 1.638 | 1 | 0.201 |
| chronic_kidney_diseases | 1.652 | 1 | 0.199 |
| BMI | 0.004 | 1 | 0.952 |
| Kcal.day | 0.458 | 1 | 0.498 |
| oral_intake_recovery | 1.408 | 1 | 0.235 |
| GLOBAL | 19.689 | 16 | 0.235 |

**Supplementary Table S10. Sensitivity analysis excluding patients with aspiration pneumonia (Asp): association between baseline Ln-LCR (continuous and quartiles) and long-term all-cause mortality.**

|  | Model 1 | |  | Model 2 | |  | Model 3 | |
| --- | --- | --- | --- | --- | --- | --- | --- | --- |
|  | HR (95% CI) | *P* value |  | HR (95% CI) | *P* value |  | HR (95% CI) | *P* value |
| **Ln-LCR continuous** | 0.72 (0.63~0.82) | <0.001 |  | 0.75 (0.65~0.86) | <0.001 |  | 0.81 (0.69~0.96) | 0.012 |
| **Ln-LCR quartiles** |  |  |  |  |  |  |  |  |
| Q1 | 1.0 [Ref] | |  | 1.0 [Ref] | |  | 1.0 [Ref] | |
| Q2 | 0.59 (0.34~1.05) | 0.072 |  | 0.60 (0.34~1.06) | 0.080 |  | 0.61 (0.32~1.15) | 0.129 |
| Q3 | 0.37 (0.20~0.69) | 0.002 |  | 0.42 (0.22~0.79) | 0.007 |  | 0.57 (0.28~1.16) | 0.121 |
| Q4 | 0.17 (0.08~0.38) | <0.001 |  | 0.20 (0.09~0.44) | <0.001 |  | 0.34 (0.14~0.83) | 0.017 |
| *P-*trend | 0.58 (0.46~0.72) | <0.001 |  | 0.61 (0.48~0.76) | <0.001 |  | 0.72 (0.55~0.93) | 0.013 |
| Model 1: no covariates were adjusted Model 2: Gender, age were adjusted Model 3: Gender, age, BMI, Kcal/day, CVD, dementia, ND, IHD, CHF, CLD, CKD, NCVC, PEG, oral intake recovery. Abbreviation: CVD, cerebrovascular diseases; ND, neuromuscular diseases; Asp, previous history of aspiration pneumonia; IHD, ischemic heart diseases; CHF, Chronic Heart Failure; CLD, Chronic Lung Diseases; CKD, Chronic Kidney Diseases; ALB, serum albumin; TLC, Total Lymphocyte Count; TC, Total Cholesterol; CRP, C-reactive Protein; NCVC, Non-tunneled Central Venous Catheters; PRG, Percutaneous Endoscopic Gastrostomy; HR hazard ratio; CI confidence interval; Ref reference. | | | | | | | | |
|  |  |  |  |  |  |  |  |  |
|  |  |  |  |  |  |  |  |  |
|  |  |  |  |  |  |  |  |  |
|  |  |  |  |  |  |  |  |  |
|  |  |  |  |  |  |  |  |  |
|  |  |  |  |  |  |  |  |  |
|  |  |  |  |  |  |  |  |  |
|  |  |  |  |  |  |  |  |  |
|  |  |  |  |  |  |  |  |  |

**Supplementary Table S11. Sensitivity analysis excluding participants with extreme C-reactive protein (CRP) values: association between baseline Ln-LCR (continuous and quartiles) and long-term all-cause mortality.**

|  | Model 1 | |  | Model 2 | |  | Model 3 | |
| --- | --- | --- | --- | --- | --- | --- | --- | --- |
|  | HR (95% CI) | *P* value |  | HR (95% CI) | *P* value |  | HR (95% CI) | *P* value |
| **Ln-LCR continuous** | 0.66 (0.54~0.8) | <0.001 |  | 0.69 (0.57~0.84) | <0.001 |  | 0.82 (0.66~1.03) | 0.088 |
| **Ln-LCR quartiles** |  |  |  |  |  |  |  |  |
| Q1 | 1.0 [Ref] | |  | 1.0 [Ref] | |  | 1.0 [Ref] | |
| Q2 | 0.72 (0.42~1.23) | 0.229 |  | 0.74 (0.43~1.27) | 0.273 |  | 0.88 (0.47~1.65) | 0.691 |
| Q3 | 0.35 (0.18~0.66) | 0.001 |  | 0.38 (0.20~0.72) | 0.003 |  | 0.52 (0.25~1.10) | 0.087 |
| Q4 | 0.25 (0.13~0.51) | <0.001 |  | 0.29 (0.14~0.58) | 0.001 |  | 0.48 (0.22~1.05) | 0.065 |
| *P-*trend | 0.62 (0.50~0.76) | <0.001 |  | 0.65 (0.52~0.80) | <0.001 |  | 0.76 (0.60~0.97) | 0.03 |
| Model 1: no covariates were adjusted Model 2: Gender, age were adjusted Model 3: Gender, age, BMI, Kcal/day, CVD, dementia, ND, Asp, IHD, CHF, CLD, CKD, NCVC, PEG, oral intake recovery. Abbreviation: CVD, cerebrovascular diseases; ND, neuromuscular diseases; Asp, previous history of aspiration pneumonia; IHD, ischemic heart diseases; CHF, Chronic Heart Failure; CLD, Chronic Lung Diseases; CKD, Chronic Kidney Diseases; ALB, serum albumin; TLC, Total Lymphocyte Count; TC, Total Cholesterol; CRP, C-reactive Protein; NCVC, Non-tunneled Central Venous Catheters; PRG, Percutaneous Endoscopic Gastrostomy; HR hazard ratio; CI confidence interval; Ref reference. | | | | | | | | |
|  |  |  |  |  |  |  |  |  |
|  |  |  |  |  |  |  |  |  |
|  |  |  |  |  |  |  |  |  |
|  |  |  |  |  |  |  |  |  |
|  |  |  |  |  |  |  |  |  |
|  |  |  |  |  |  |  |  |  |
|  |  |  |  |  |  |  |  |  |
|  |  |  |  |  |  |  |  |  |
|  |  |  |  |  |  |  |  |  |


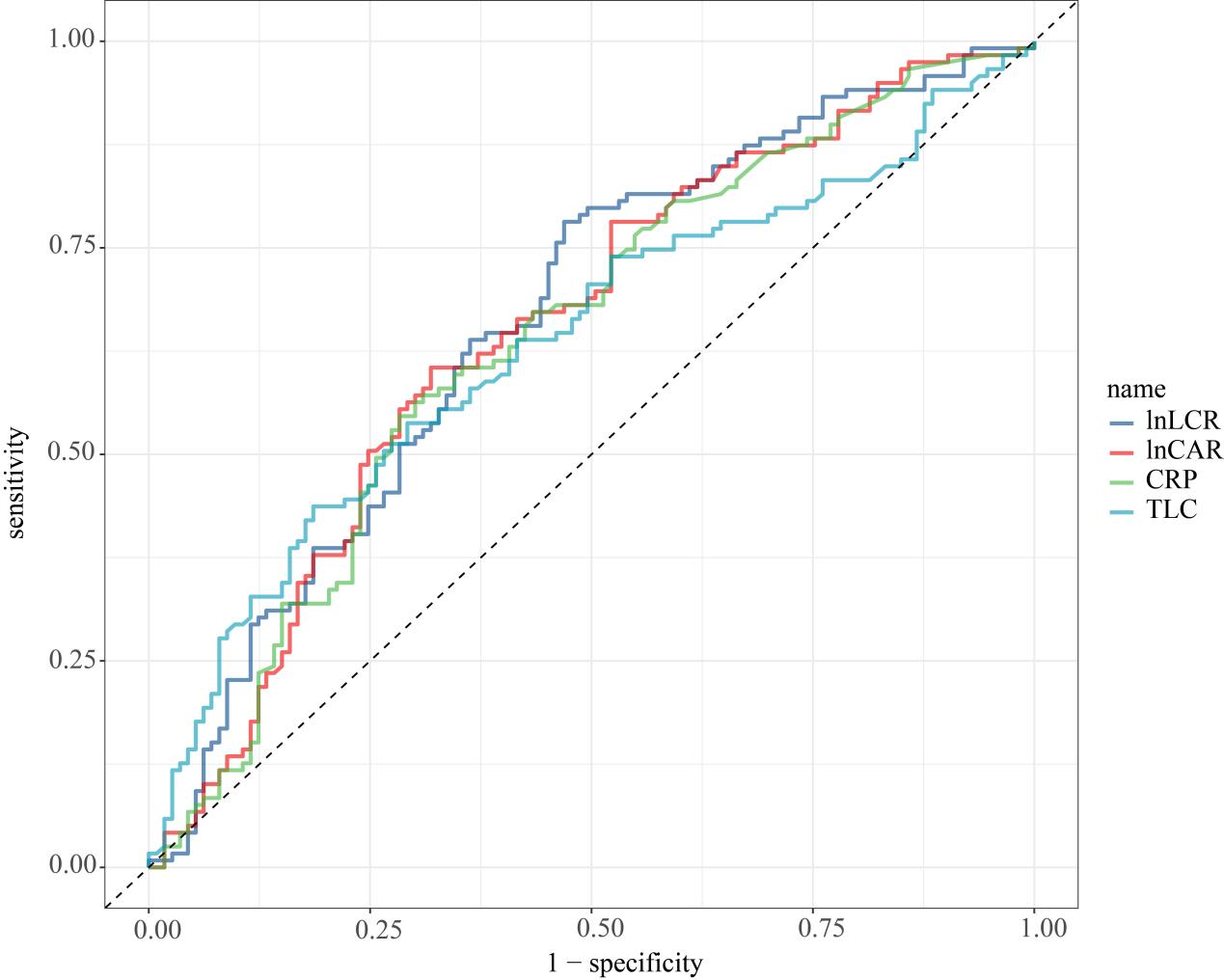


**Figure S1.** ROC curves comparing four markers for predicting mortality
